# Multidrug-resistant non-typhoidal *Salmonella* of public health significance recovered from migratory birds in Bangladesh

**DOI:** 10.3389/fmicb.2023.1162657

**Published:** 2023-05-15

**Authors:** Roderick M. Card, Thomas Chisnall, Ruhena Begum, Md Samun Sarker, Muhammad Sazzad Hossain, Md Shahjalal Sagor, Mohammad Asheak Mahmud, A. S. M. Ashab Uddin, Md Rezaul Karim, Johanna F. Lindahl, Mohammed Abdus Samad

**Affiliations:** ^1^Animal and Plant Health Agency, New Haw, Addlestone, United Kingdom; ^2^Antimicrobial Resistance Action Center (ARAC), Animal Health Research Division, Bangladesh Livestock Research Institute, Savar, Bangladesh; ^3^Department of Clinical Sciences, Swedish University of Agricultural Sciences, Uppsala, Sweden

**Keywords:** *Salmonella*, migratory birds, multidrug resistance, antimicrobial resistance, Bangladesh

## Abstract

Non-typhoidal *Salmonella* provides an exemplar for the One Health approach as it encompasses public and animal health, food safety, and environmental considerations. The contribution of environmental aspects is currently less well-defined. The purpose of this study was to determine the carriage occurrence of non-typhoidal *Salmonella* in migratory birds in Bangladesh and assess the potential significance to public and animal health. Cloacal swabs (*N* = 453) were collected in the years 2018–2020 from Tanguar and Hakaluki Haors, important wetland ecosystems in Northeastern Bangladesh. The prevalence of *Salmonella* was 13.5% (61 positive swabs). Classical serotyping identified six serovars: *Salmonella enterica* subsp. *enterica* serovars Perth, Kentucky, Albany, Infantis, Weltevreden, and Brancaster. Resistance towards 14 antimicrobials was assessed by broth microdilution minimum inhibitory concentration determination and the antimicrobial resistance (AMR) genotype established by whole-genome sequencing. *S.* Perth and *S.* Weltevreden isolates were susceptible and harbored no acquired AMR genes. Isolates from the remaining serovars were multidrug resistant, commonly possessing resistance to tetracycline, ampicillin, chloramphenicol, sulfamethoxazole, trimethoprim, and ciprofloxacin. *Salmonella* resistant to ciprofloxacin meets WHO criteria for priority pathogens. There was excellent concordance between resistance phenotype and the presence of corresponding AMR genes, many of which reside on *Salmonella* Genomic Islands. High-level ciprofloxacin resistance correlated with the presence of mutations in the chromosomal *gyrB* and/or *parC* genes. The *S.* Kentucky isolates were ST198, a widely distributed multidrug-resistant lineage reported in humans and animals, and constituting an ongoing risk to public health worldwide. We have demonstrated that multidrug-resistant non-typhoidal *Salmonella* of public health significance can be recovered from migratory birds. A potential for risk can manifest through direct interaction, transmission to food-producing livestock on farms, and dissemination via the long range migratory movements of birds. Risks can be mitigated by measures including continued surveillance and implementation of good farm biosecurity practices.

## Introduction

*Salmonella enterica* is a zoonotic pathogen of significant public and animal health concern worldwide (Majowicz et al., 2010). The species is highly diverse, having over 2,600 serovars (Grimont and Weill, 2007), which have been divided into typhoidal and non-typhoidal *Salmonella* (NTS) serovars. Non-typhoidal *Salmonella* (NTS) can cause gastroenteritis that is generally self-limiting in humans, but can lead to an invasive infection that presents a greater risk to health and may require antimicrobial treatment (Acheson and Hohmann, 2001). The number of foodborne illnesses and deaths caused by NTS globally in 2010 has been estimated at over 78 million and >59,000, respectively, (Havelaar et al., 2015). The prevalence of NTS serovars differs, but in any given country, most human isolates (>90%) will be from about 30 serovars (Grimont and Weill, 2007). Poultry and poultry products are a common source of human infection by NTS, and important *Salmonella enterica* subsp. *enterica* serovars include *S.* Typhimurium, *S.* Enteritidis, *S.* Kentucky, and *S.* Infantis, among others (Koutsoumanis et al., 2019).

Antimicrobial resistance (AMR) in *Salmonella* is of considerable concern as it limits therapeutic options and increases the risk of treatment failure. Furthermore, multidrug resistance (resistance to ≥3 antimicrobial classes) has been associated with more serious disease in people (Parisi et al., 2018). Traditional first-line antibiotics include ampicillin, chloramphenicol, and trimethoprim/sulfamethoxazole (Crump et al., 2015). The high prevalence of resistance in *Salmonella* to these antimicrobials has led to increased use of critically important antimicrobials such as ciprofloxacin (fluoroquinolone), ceftriaxone (third-generation cephalosporin), and alternatively azithromycin (macrolide; Crump et al., 2015). *Salmonella* with resistance to fluoroquinolones, carbapenems, or third generation cephalosporins have been listed as high-priority pathogens by the World Health Organization (Tacconelli et al., 2018).

*Salmonella* has been found in resident and migratory free-living birds in many countries (Tizard, 2004; Beleza et al., 2020). Wild birds can act as vectors that introduce pathogens including *Salmonella* onto farms, necessitating the need for appropriate biosecurity (De Lucia et al., 2018; Koutsoumanis et al., 2019). The role of resident and migratory birds as a reservoir and disseminator of *Salmonella* (Tizard, 2004; De Lucia et al., 2018; Beleza et al., 2020; Lin et al., 2020), AMR (Lin et al., 2020; Islam et al., 2021, 2022), and viruses (Gilbert et al., 2010; Bi et al., 2015) is widely recognized. The aggregation of large mixed species groups of wild birds can facilitate the spread of *Salmonella*, AMR, and other pathogens. The long-range migratory movements of birds frequently span countries and continents, providing significant potential for the dispersal to other areas of *Salmonella* and AMR harbored therein.

Bangladesh lies in the path of two migratory flyways, the East Asian–Australasian Flyway and the Central Asian Flyway. Birds migrate between breeding grounds in Northern regions and their overwintering quarters in the South. Migratory birds either overwinter in Bangladesh or pass through as part of their annual migration. Haors, diverse wetland ecosystems in Northeastern Bangladesh, provide important habitats for resident and migratory birds. Two important Haors are Tanguar Haor and Hakaluki Haor located in the sub-districts of Tahirpur and Fenchuganj under the districts of Sunamganj and Sylhet, respectively. Tanguar Haor is a wetland of international importance listed as a Ramsar site Under the Convention on Wetlands^1^ and was declared an Ecologically Critical Area in 1999 by the Bangladesh government. Tanguar Haor provides a habitat for 98 migratory bird species and up to ~40,000 migratory waterfowl converge on the area in the northern winter months.^2^ The Haor also supports the livelihoods of many people, potentially bringing them into contact with wildlife, and is an important inland fisheries system with rich breeding grounds for freshwater fish. Studies of rivers and surface water in Bangladesh have shown the presence of *Salmonella* (Faridullah et al., 2022; Yasmin et al., 2023).

The occurrence of *Salmonella* in poultry and poultry products in Bangladesh has been well documented and includes serovars of public health significance, such as *S.* Typhimurium, *S.* Enteritidis, and *S.* Kentucky (Barua et al., 2012, 2013; Makendi et al., 2016). The poultry-adapted serovars *S.* Gallinarum and *S.* Gallinarum biovar Pullorum, which cause fowl typhoid and Pullorum disease, respectively, have also been reported in Bangladesh (Parvej et al., 2016). *Salmonella* has also been detected in shrimp and on shrimp farms in Bangladesh (Hossain et al., 2013; Faridullah et al., 2016). Furthermore, *Salmonella* has been described in migratory (Islam et al., 2021) and resident (Faruq et al., 2016) wild birds in Bangladesh. Both studies reported multidrug-resistant *Salmonella*, but neither described the serovar or the AMR genotype. Determination of serovar, occurrence of AMR, and genotype can allow the assessment of the significance of isolates to public and animal health. The importance of a One Health approach to tackling AMR and food-borne zoonoses such as *Salmonella* in a manner that encompasses wildlife has been highlighted in a systematic review from the Bangladesh perspective (Khan et al., 2020).

The prevalence and diversity in migratory birds of *Salmonella* and its AMR in Bangladesh remain poorly defined. To address this evidence gap, we undertook a study to assess the occurrence of *Salmonella* and AMR in migratory wild birds at Haors in Bangladesh over 3 years. To help assess potential for risk to livestock and people, we determined the serovar and antimicrobial susceptibilities of isolates obtained and used whole-genome sequencing (WGS) to define resistance gene carriage.

## Materials and methods

### Study design and location

**Figure 1:**
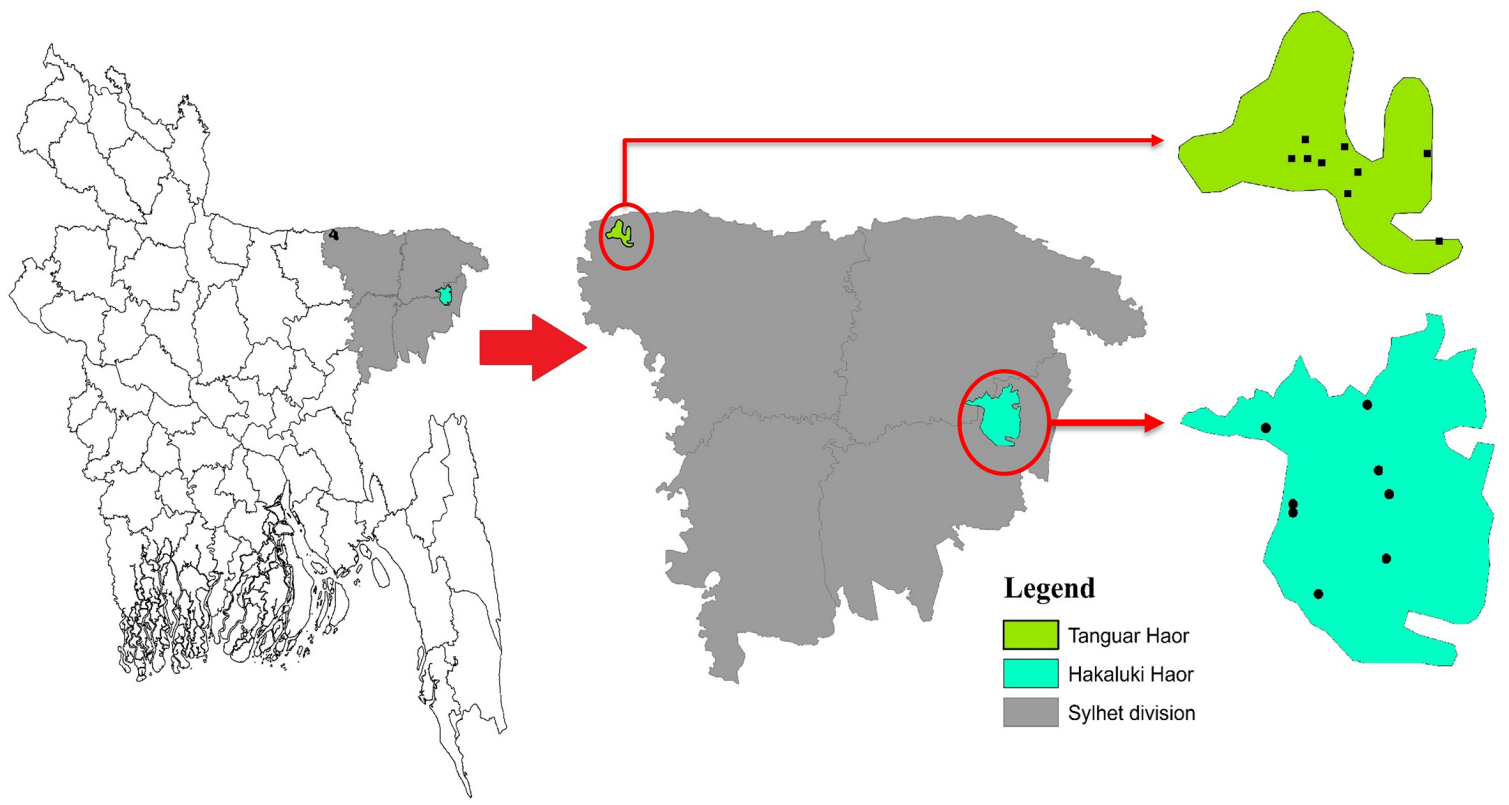
Sampling area of Tanguar Haor and Hakaluki Haor in Bangladesh.

During three consecutive years from 2018 to 2020, cloacal samples of different migratory birds were collected from Tanguar Haor and Hakaluki Haor in Bangladesh (Figure 1). Hakaluki and Tanguar Haors are in Northeastern Bangladesh and were chosen as the study sites as every year these wetlands intake thousands of migratory birds (Hassan et al., 2020). The birds were captured alive by expert trappers using the nets during the winter period of Bangladesh (November–December and January–February). No anesthesia, euthanasia, or animal sacrifice was conducted in this study, and precautionary measures were taken to avoid any potential harm to the birds. Out of 453 samples, 109 were collected in 2018, 133 in 2019, and 211 in 2020. Most samples (419) were collected from Tanguar Haor during three consecutive years, and 34 samples were collected from Hakaluki Haor in 2018. The number of migratory birds sampled varied depending on availability and capability to capture by expert trappers in different years and locations. During 2018, there was access to both Tanguar Haor and Hakaluki Haor, but from 2019 onwards, access to Hakaluki Haor was restricted by the local authority for conservation purposes. The samples were collected from 10 species of migratory birds, those locally called Thila (*Mareca strepera*), Peri (*Aythya ferina*), Lenja Has (*Bucephala clangula*), Bali Has (*Branta canadensis*), Oikko (*Unknown*), Kalo Kura/Ramer Kura (*Fulica atra*), Boral (*Aythya nyroca*), Moulovi (*Netta rufina*), Matarangi (*Mareca penelope*), Oda (*Unknown*), and Khaium (*Unknown*). The details of the sampling pattern are shown in Table 1.

**Table 1:** Geographic and demographic information of samples.

Year	Haor	Samples per species of migratory birds											Total samples
		*Mareca strepera*	*Aythya ferina*	*Bucephala clangula*	*Branta canadensis*	Oikko (Unknown)*	*Fulica atra*	*Aythya nyroca*	*Netta rufina*	*Mareca penelope*	Oda (Unknown)*	Khaium (Unknown)*	
2018	Tanguar	15	9	10	0	3	15	7	3	3	8	2	75
	Hakaluki	6	8	6	0	0	5	0	0	0	9	0	34
2019	Tanguar	11	13	14	3	4	38	24	17	7	2	0	133
2020	Tanguar	22	32	34	0	3	47	50	0	3	9	11	211

Bird species with an asterisk (*) were identified by the local Bangladeshi common name only.

### Ethical approval

The ethical approval was received from the Ethical Committee of the Animal Health Research Division at the Bangladesh Livestock Research Institute, Dhaka, Bangladesh (ARAC: 10/1/2018:01).

### Isolation and identification of *Salmonella* species

Isolation and identification of *Salmonella* were carried out according to the ISO guidelines as follows: cloacal swabs were pre-enriched in buffered peptone water (BPW; Oxoid, UK) with a dilution rate of 1:10 then incubated aerobically at 37°C for 18–24 h. After enrichment, 0.1 ml of sample was then inoculated at three different locations on Modified Semisolid Rappaport Vassiliadis (MSRV; Oxoid, UK) agar medium and incubated at 41.5°C for 20–24 h. Furthermore, MSRV was plated onto xylose lysine deoxycholate (XLD; Oxoid, UK) and MacConkey agar plate and incubated overnight at 37°C (ISO, 6579: 2002; Foley et al., 2008). Characteristic black-centered colonies with reddish zones on the XLD and non-lactose-fermenting colonies on the MacConkey were selected and sub-cultured on nutrient agar medium and performed biochemical testing [triple sugar iron (TSI), motile indole urea (MIU)], catalase, and oxidase. Final confirmation was performed with a Vitek-2 compact analyzer (bioMérieux, Marcyl’Étoile, France), followed by the polymerase chain reaction. The antigenic formula of each strain was determined using standardized methods (Shipp and Rowe, 1980), and serovar was assigned according to the White–Kauffmann–Le Minor scheme (Grimont and Weill, 2007). The proportion of positive samples by year and serovar (see results) and the 95% confidence interval of the proportions were calculated with GraphPad^3^ using the modified Wald method for confidence intervals.

### Antimicrobial susceptibility testing

Antimicrobial susceptibility testing was performed by broth microdilution minimum inhibitory concentration (MIC) determination using commercial plates (Sensititre™ EU Surveillance Salmonella/*Escherichia coli* EUVSEC plate, Thermo Fisher Scientific, Basingstoke, UK), according to the manufacturer’s instructions. Briefly, a suspension of each isolate was prepared to a density of 0.5 McFarland in 5 ml of demineralized water and 10 μl transferred to 11 ml of Mueller Hinton broth to obtain a target inoculum density of between 1 × 10^5^ and 1 × 10^6^ CFU/ml. Fifty microlitres were dispensed into each well of the microtitre plate and incubated at 35–37°C for 18 to 24 h. Fourteen antimicrobials were tested in this manner (sulfamethoxazole, trimethoprim, ciprofloxacin, tetracycline, tigecycline, azithromycin, nalidixic acid, ampicillin, cefotaxime, ceftazidime, meropenem, chloramphenicol, colistin, and gentamicin), and the MICs were recorded as the lowest concentration preventing visible growth. *Escherichia coli* NCTC 12241 (ATCC 25922) was used as the control strain. Susceptibility was assessed using epidemiological cutoff (ECOFF) values (EUCAST, 2020), except for azithromycin, colistin, and tigecycline for which the human clinical breakpoints proposed by EFSA were employed (ECDC, 2016; as ECOFF values were not available). Isolates with non-wild type susceptibilities have been termed “microbiologically resistant,” although it is recognized this is not necessarily synonymous with clinical resistance. Isolates were defined as multidrug resistant when non-wild type for three or more classes of antimicrobial (Schwarz et al., 2010).

### Whole-genome sequencing

Deoxyribonucleic acid (DNA) extracts were prepared from overnight Mueller Hinton broth cultures and extracted with the MagMAX™ CORE extraction kit (Thermo Fisher Scientific, Basingstoke, UK) using the semi-automated KingFisher Flex system (Thermo Fisher Scientific, Basingstoke, UK) according to the manufacturer’s instructions. Extracted DNA was processed for whole-genome sequencing using the Illumina HiSeq platform. The resulting raw sequences were analyzed using the Nullarbor 2 pipeline,^4^ using as reference the published genome *S.* Kentucky strain 201001922 [accession number CP028357; (Hawkey et al., 2019)], Spades for genome assembly [version 3.14.1; (Bankevich et al., 2012)], and Prokka for annotation [version 1.14.6; (Seemann, 2014)]. The presence of genes and point mutations conferring AMR was assessed using AMRFinderPlus (Feldgarden et al., 2019). The sequence type (ST) was determined with MLST (version 2.19.0; https://github.com/tseemann/mlst) using the PubMLST database (Jolley et al., 2018). Virulence gene presence was assessed using Abricate^5^ and the virulence factor database (Chen et al., 2016) in Nullarbor. The presence of heavy metal resistance genes was assessed using AMRFinderPlus.

The relatedness of the *S.* Perth isolates (see results) was assessed using Snippy (version 4.6.0) and SNPdist (version 0.8.2), with the reference genome *S.* Perth strain FDA363901 (Accession GCA_006407535.1) obtained from a Bangladesh Pabda fish. Two additional *S.* Perth isolates from Bangladesh Pabda fish and three human isolates from the UK were included in this comparison (Supplementary Table S2). These isolates represented all publicly available *S.* Perth genomes in EnteroBase (accessed 21 June 2022; Zhou et al., 2020). The relatedness of the *S.* Kentucky isolates (see results) was assessed in the same manner, using *S.* Kentucky strain 201001922 as a reference genome (accession number GCA_003030965.1). This analysis included the two published genomes from Bangladesh isolates (poultry strain K_50, accession number SRS3092506, and a human strain deposited in EnteroBase as SAL_LB9505AA) and 115 additional isolates from Asia and the Middle East (Supplementary Table S3), to enable an assessment of relatedness towards *S.* Kentucky from Bangladesh and other countries. Phylogenetic trees were produced using the core genome single-nucleotide polymorphism (SNP) alignments using default settings, and maximum-likelihood trees were bootstrapped 200 times using RAxML-NG (version 1.1.0; Kozlov et al., 2019). Trees were viewed and annotated in Interactive Tree Of Life (version 6.6; Letunic and Bork, 2016).

The whole-genome sequences were deposited in the National Center for Biotechnology Information (NCBI) National Library of Medicine under BioProject accession number PRJNA933150.

## Results

### Prevalence of *Salmonella* in migratory birds

To assess the carriage of *Salmonella* in migratory birds, 453 cloacal samples were analyzed and 61 cloacal samples were found to be positive for *Salmonella* species, giving an overall prevalence of 13.5% (Table 2). The prevalence was 33.02, 14.28, and 2.84% in the years 2018, 2019, and 2020, respectively. In 2018, sampling was undertaken at Tanguar and Hakaluki Haors, and *Salmonella* prevalence was 41% (31/75) and 16% (5/34), respectively.

**Table 2:** Prevalence of *Salmonella* by year.

Year	2018	2019	2020	Total
Field samples tested	109	133	211	453
Positive samples	36	19	6	61
Prevalence	33.0%	14.3%	2.8%	13.5%
Confidence interval (95% CI)	25.0–42.3%	9.3–21.3	1.2–6.2%	10.6–16.9%

Six *Salmonella* serovars were identified in the 61 isolates (Table 3; Supplementary Table S1). The most common was *S.* Perth, which comprised 62% (38/61) of all isolates and was detected in each of the three study years. *S.* Infantis was the only other serovar detected in more than 1 year, in 2018 and 2020, with an overall prevalence of 7% (4/61). The remaining four serovars were detected in 1 year only, including 12 *S.* Kentucky in 2018 (Table 3). In 2018, three serovars were detected at both Haors: *S.* Perth, *S.* Kentucky, and *S.* Albany (Supplementary Table S1).

**Table 3:** Distribution of different *Salmonella* serovars by year.

Serovar	2018	2019	2020	Total	Overall prevalence	Confidence interval (95% CI)
Perth	18	19	1	38	62.3%	49.7–73.4%
Kentucky	12	0	0	12	19.7%	11.5–31.5%
Albany	5	0	0	5	8.2%	3.8–18.2%
Infantis	1	0	3	4	6.6%	2.1–16.1%
Weltevreden	0	0	1	1	1.6%	<0.1–9.6%
Brancaster	0	0	1	1	1.6%	<0.1–9.6%

Eight species of migratory birds were positive for *Salmonella*, with the majority (49/61; 80%) from four migratory species that overwinter in Bangladesh: *Aythya farina* (*n* = 10), *A. nyroca* (*n* = 12), *Fulica atra* (*n* = 12), and *Mareca strepera* (*n* = 15; Supplementary Table S1). There was no apparent association between bird species and the presence of *Salmonella*. Low numbers of birds were affected by some serovars, and the most prevalent serovar affected the highest number of different bird species. *S.* Perth was isolated from eight bird species, *S.* Kentucky from four bird species, *S.* Infantis was isolated from three species, and *S.* Albany was isolated from two species (Supplementary Table S1). Three positive samples were obtained from birds for which only the common name was known.

### Antimicrobial resistance and carriage of AMR genes

Antimicrobial resistance was assessed using phenotypic (broth microdilution) and genotypic (WGS) approaches, and there was excellent correspondence between resistances and the presence of AMR determinants (Supplementary Table S1). No isolates were resistant to azithromycin, colistin, cefotaxime, ceftazidime, or meropenem. Indeed, the *S.* Perth (*n* = 38) and *S.* Weltevreden (*n* = 1) isolates were fully susceptible to all 14 antimicrobials tested. No AMR genes were detected in *S.* Weltevreden, whereas the *S.* Perth isolates harbored the chromosomally encoded fosfomycin resistance gene *fosA7.7* (susceptibility towards this antimicrobial was not tested). The five *S.* Albany isolates harbored the SGI-1 associated genes *bla*_CARB-2_, *floR*, *sul1*, *tet*(G), and *dfrA1*, and were correspondingly resistant to ampicillin, phenicols, sulfamethoxazole, tetracycline, and trimethoprim. Additionally, Albany isolates were resistant to the quinolone antimicrobials ciprofloxacin and nalidixic acid, and carried a mutation in the *gyrA* gene giving the D87N substitution in the amino acid sequence associated with quinolone resistance. The *S.* Brancaster isolate was also multidrug resistant, with non-wild type susceptibilities for ampicillin (*bla*_TEM-176_), phenicols (*floR*), ciprofloxacin (*qnrS*), tetracycline (*tet*(A)), and trimethoprim (*dfrA14*); it also harbored the resistance gene *aph*(*3′*)*-Ia* (susceptibility towards this antimicrobial was not tested). The *S.* Kentucky isolates harbored *bla*_TEM-1_, *aac*(*3*)*-Id*, *sul1*, and *tet*(A) and were resistant to ampicillin, gentamicin, sulfamethoxazole, and tetracycline. They were also resistant to ciprofloxacin and nalidixic acid and harbored mutations *gyrA* (S83F), *gyrA* (D87Y), and *parC* (S80I) associated with resistance to quinolones. Additionally, the streptomycin resistance gene *aadA7* was in all isolates and the kanamycin resistance gene *aph*(*3′*)*-Ia* was present in two isolates (susceptibility towards these two antimicrobials was not tested). All four *S.* Infantis isolates were resistant to sulfamethoxazole (*sul1*), trimethoprim (*dfrA14*), ciprofloxacin, and nalidixic acid (*gyrA* (D87Y)). Three *S.* Infantis isolates, all from 2020, were additionally resistant to ampicillin (*bla*_TEM-1_), chloramphenicol (*cmlA5*), gentamicin [*ant*(*2″*)*-Ia*], and tetracycline [*tet*(A)].

### Sequence type

Isolate serovar was closely associated with sequence type (ST): *S.* Albany (ST292), *S.* Brancaster (ST2133), *S.* Infantis (ST32), **S.* Kentucky* (ST198), *S.* Perth (ST2245), and *S.* Weltevreden (ST365; Supplementary Table S1).

**Figure 2:**
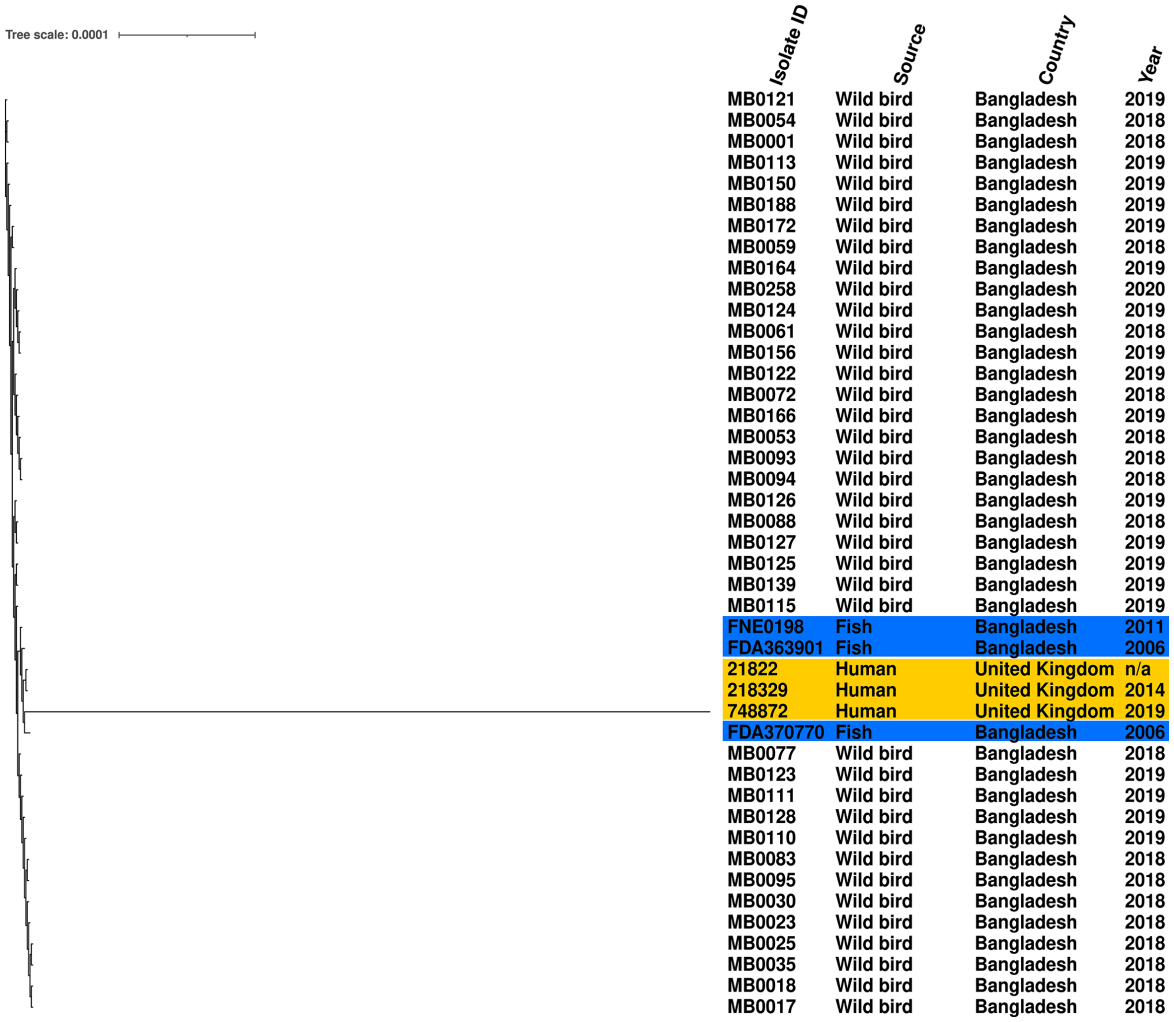
Maximum-likelihood phylogenetic tree generated from core genome single-nucleotide polymorphisms of *S.* Perth isolates. Blue highlight indicates published genomes from Bangladesh fish, and yellow highlight indicates published genomes from UK humans. Country, source, and year are given for each isolate. Reference was *S.* Perth strain FDA363901.

Given the high prevalence of *S.* Perth, its persistence across 3 years, detection at two Haors, and conserved ST, we further examined the genomic diversity of these isolates by comparison to a reference genome. The phylogenetic tree demonstrates that all *S.* Perth were closely related, except one isolate from a human in the UK (Figure 2). Indeed, in the core genome spanning 4,706,256 bp, only 2,282 were variable single-nucleotide polymorphisms (SNPs). The migratory bird isolates had a 0–7 SNPs difference from each other, compared to 15–19 SNPs difference with the reference isolate, which was obtained from a Bangladesh fish (Supplementary Table S2). There was also high sequence identity with a second isolate from a Bangladesh fish (12–18 SNPs difference) and with two UK human isolates (21–26 SNPs difference); there was considerably greater SNPs difference in comparison to the third UK human isolate (2,188–2,275 SNPs; Supplementary Table S2).

**Figure 3:**
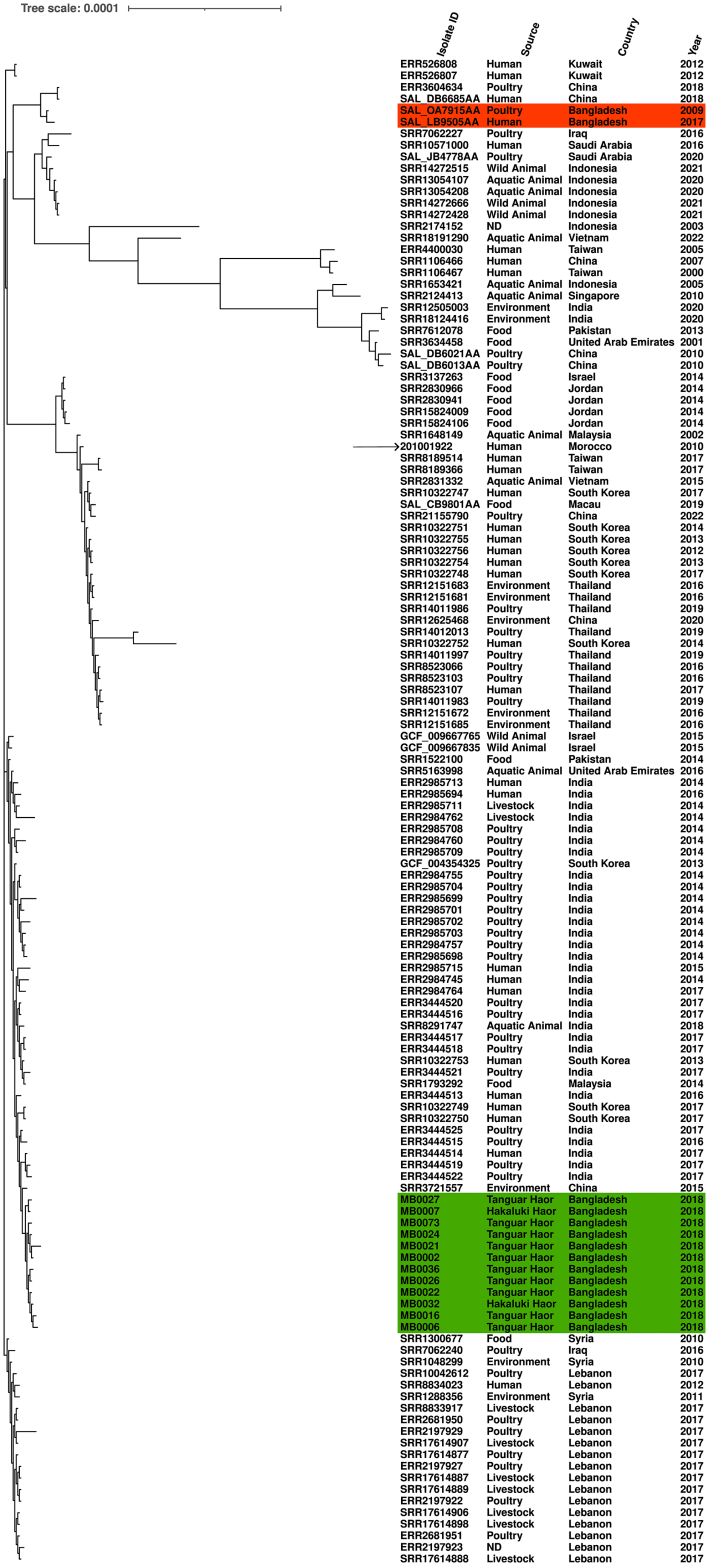
Maximum-likelihood phylogenetic tree generated from core genome single-nucleotide polymorphisms of *S.* Kentucky isolates. Migratory bird isolates indicate in green and previously published genomes from Bangladesh are shown in orange. Country, source, and year are given for each isolate. Reference was *S.* Kentucky strain 201001922 (indicated by arrow).

Another of the serovars further examined was *S.* Kentucky, which was identified from isolates from 2018 and detected in two Haors. We compared the genomic diversity of these isolates to the reference genome *S.* Kentucky strain 201001922 (accession number CP028357.1), alongside 117 *S.* Kentucky strains isolated from across Asia. The migratory bird isolates clustered in a single sub-clade, which included isolates from humans (India and South Korea), poultry (India), or environmental samples (China; Figure 3). The two previously published *S.* Kentucky genomes from Bangladesh did not reside in this sub-clade (Figure 3). The core genome spanned 4,858,671 bp of which 292 were SNPs (Supplementary Table S3). The migratory bird isolates from this study had 4–46 SNPs difference from each other, compared to 222–262 SNPs difference with the reference isolate. The migratory bird isolates from this study had 125–169 SNPs differences from the two *S.* Kentucky strains previously isolated from Bangladesh, one from poultry and one from a human isolate (Supplementary Table S3).

### Virulence and heavy metal tolerance genes

A total of 115 virulence factors were identified in the 61 *Salmonella* isolates (Supplementary Table S4), and the majority were shared by most serovars, including fimbriae (*fim* genes), adhesins (*misL*), invasins (*invA*), effectors (*avrA*), secretion systems (*prgJ*), and a macrophage inducible gene (*mig-14*). We also investigated the presence of heavy metal resistance genes (Supplementary Table S4). Heavy metal resistance genes from the *pco* (copper) and *sil* (copper/silver) operons were present in the *S.* Brancaster isolate. The *S.* Kentucky and Infantis isolates carried genes from the *mer* (mercury) operon; Infantis isolates additionally harbored *arsR* (arsenic resistance).

## Discussion

In this study, the overall prevalence of *Salmonella* in migratory birds in Bangladesh was 13.5%. This is similar to the prevalence of 21.2% reported in Bangladesh by a previous study (Saiful Islam et al., 2021); however, this earlier study was limited by a low sample size (*n* = 66) and tested freshly deposited fecal material without correlation to bird species. Another study of Bangladesh wild birds reported a *Salmonella* prevalence of 65% in *Corvus splendens* (house crow) and 67% in *Gracupica contra* (Asian pied starling), although neither bird is migratory (Faruq et al., 2016). We observed a reduction in *Salmonella* prevalence between 2018 and 2020, from 33.0 to 2.8%, despite increased sampling efforts over this period. This may reflect fluctuations in prevalence and changes in the *Salmonella* population resident in migratory birds over time. Changes in *Salmonella* prevalence over time in wild bird populations are currently poorly defined. Studies in other countries have sampled across multiple years but reported *Salmonella* prevalence in the aggregate, such as South Korea (0.93%), Singapore (0.99%), Uganda (4.3%), and Poland (6.4%; Krawiec et al., 2015; Afema and Sischo, 2016; Aung et al., 2019; Wei et al., 2020).

We identified six different *Salmonella* serovars in the birds, of which *S.* Albany, *S.* Kentucky, and *S.* Infantis have been described in wild birds previously (Beleza et al., 2020). Unexpectantly, the most common serovar was *S.* Perth. This serovar has been rarely reported, with only six genomes available in EnteroBase (accessed 21 June 2022; Zhou et al., 2020), including three UK human isolates and three isolates from Bangladesh freshwater fish. The high sequence identity observed within our wild bird isolates (0–7 SNPs in the core genome) meets the proposed relatedness threshold criteria (Schurch et al., 2018) to indicate that these isolates are likely to be representatives of a single clone, which had therefore been isolated from multiple birds in different years and at different Haors. Indeed, this was the only serovar detected every year, and may indicate a potential for a persistent environmental presence by this clone. The isolates also had high sequence identity to two *S.* Perth isolates from Bangladesh fish and two UK human isolates; however, the number of SNPs difference marginally exceeded the relatedness threshold to be classified as a clone. Nevertheless, the potential for an association of this rare serovar between migratory birds and freshwater fish in Bangladesh is noteworthy. Indeed, many of the bird species from which *S.* Perth was isolated are omnivorous and include small fish in their diet (Birdlife, 2023). Furthermore, *Salmonella* have been detected in rivers and surface water in Bangladesh, although serovars were not reported (Faridullah et al., 2022; Yasmin et al., 2023). This may suggest an ecological aspect in which fish and piscivorous birds share the same *Salmonella*; however, investigation of fish and other environmental reservoirs such as water and soil was beyond the scope of this study. This possibility of an ecological aspect may warrant further investigation in future as it could indicate a source and potential route for the infection of humans through the consumption of food. Importantly, the *S.* Perth isolates from wild birds harbored no acquired AMR genes.

In contrast, 12 *S.* Kentucky isolates were multidrug-resistant and harbored genes conferring resistance to antimicrobials, disinfectants, and heavy metals; these genes have previously been reported to reside on SGI-1 in this serovar (Hawkey et al., 2019). All isolates were ST198, a globally distributed multidrug-resistant lineage reported in humans and animals, constituting an ongoing risk to public health worldwide (Hawkey et al., 2019; Mahindroo et al., 2019; Lei et al., 2020). We believe this is the first report of the *S.* Kentucky ST198 lineage in migratory birds, although (Afema and Sischo, 2016) described multidrug-resistant *S.* Kentucky isolates from wild birds in Uganda but did not report the ST. The isolates were not closely related to the two published Bangladesh genomes from humans and poultry but did reside in a sub-clade that included isolates from China, India, and South Korea. Migratory bird isolates MB0002 and MB0024 met the proposed relatedness threshold criteria of <=5 SNPs in the core genome to be considered clones; both samples were collected at Tanguar Haor, MB0002 in January 2018 and MB0024 in December 2018. Poultry and poultry products are considered a major route for infection of people by this lineage, and *S.* Kentucky has been reported in poultry in Bangladesh (Barua et al., 2012, 2013; Hawkey et al., 2019). ST198 has also been reported in poultry from India (Mahindroo et al., 2019) and China (Lei et al., 2020), both countries on migratory flyways which pass through Bangladesh.

We detected *S.* Infantis ST32 in 2018 and 2020 and noted different resistance phenotypes and AMR gene carriage between the 2 years. *S.* Infantis is of global public health importance as it frequently causes foodborne illness in people and is a commonly isolated serovar from poultry (Koutsoumanis et al., 2019). Importantly, these isolates did not carry the extended-spectrum β-lactamase gene *bla*_CTX-M-65_, associated with pESI-like plasmids (Alba et al., 2020), which has been detected in *S.* Infantis in many countries, including in wild owls from Chile (Fuentes-Castillo et al., 2019).

*S.* Albany has been emerging as a commonly isolated multidrug-resistant serovar from poultry, slaughterhouses, and human cases in several Asian countries (Hong et al., 2021; Wei et al., 2021). Here we describe multidrug-resistant *S.* Albany in Bangladesh migratory birds, harboring the same resistance phenotype and AMR determinants as reported in these countries, including the SGI-F associated genes *bla*_CARB-2_, *floR*, *sul1*, *tet*(G), and *dfrA1*. The emergence of *S.* Albany in poultry in South Korea has been hypothesized to have resulted from international travel or imported meat (Wei et al., 2021), but migratory birds may have a role in the dissemination of this serovar to poultry and between countries as Bangladesh and South Korea both lie on the East Asian–Australasian Flyway.

Similarly, *S.* Weltevreden has emerged as an important foodborne pathogen causing gastroenteritis, particularly in South-East Asian countries, and has been found in Bangladesh poultry (Barua et al., 2013; Makendi et al., 2016). Antimicrobial susceptibility and the carriage of AMR genes are generally rare in this serovar (Aarestrup et al., 2003), and the single isolate we obtained from migratory birds harbored no AMR genes and was fully susceptible to the 14 antimicrobials tested.

A single *S.* Brancaster isolate was obtained from migratory birds. This serovar has been associated with human infection in Nigeria (Ifeanyi et al., 2014) and Senegal (Cardinale et al., 2005), and detected in poultry meat in Singapore, Malaysia, and Taiwan (Chin et al., 2017; Zwe et al., 2020; Chang et al., 2022). Multidrug resistance is common and associated with a core set of AMR genes, which were present in the wild bird isolates, comprising *floR* (phenicol), *qnrS* (quinolone), *bla*_TEM-176_ (ampicillin), *tet*(A; tetracycline), *dfrA14* (trimethoprim), and *aph*(*3′*)*-Ia* (aminoglycosides; Chin et al., 2017, Zwe et al., 2020).

Noteworthily, we did not detect *S.* Typhimurium or *S*. Enteritidis, major serovars associated with human infection. *S.* Gallinarum and *S.* Gallinarum biovar Pullorum, which cause fowl typhoid and Pullorum disease were outside the scope of this study and not detected, as MRSV is unsuitable for the detection of non-motile S*almonella* bacteria (Poppe et al., 2004). These serovars have been described in Bangladesh poultry (Barua et al., 2013; Parvej et al., 2016; Siddiky et al., 2021) and wild birds in other countries (Beleza et al., 2020).

In conclusion, we have shown that a diversity of *Salmonella* serovars can be recovered from migratory birds in Bangladesh that can pose a threat to public health. *Salmonella* was detected every year for 3 years in a proportion of migratory birds. Many serovars were resistant to ampicillin, chloramphenicol, and trimethoprim/sulfamethoxazole and were multidrug resistant, which has been associated with more serious diseases in people (Parisi et al., 2018). Four of six serovars were resistant to the fluoroquinolone ciprofloxacin, meeting WHO criteria for classification as a priority pathogen (Tacconelli et al., 2018). However, all isolates in this study were susceptible to the critically important antimicrobials azithromycin, cefotaxime, ceftazidime, and meropenem (Anonymous, 2019). Exposure to *Salmonella* via wild birds can present a route to human infection, which may be an important consideration for those living and working in the Haors. Infected wild birds can present a risk of *Salmonella* infection to food-producing animals through interaction with the livestock and farm premises. Similarly, interaction with livestock and farm premises provides opportunities for exposure of wild birds to *Salmonella*. Inadequate farm biosecurity and inappropriate antimicrobial use, as has been reported for poultry farms in Bangladesh (Imam et al., 2021; Siddiky et al., 2022), heightens the risk of multidrug-resistant *Salmonella* becoming introduced and established on farms. A further concern is that the long migratory routes of the birds present a risk for the dissemination of *Salmonella* between countries, as has been observed for highly pathogenic avian influenza (Gilbert et al., 2010; Bi et al., 2015). Migratory birds could, therefore, play a role in the spreading of *Salmonella* serovars that subsequently emerge at high prevalence in livestock. The risk wild birds can present to livestock and people, therefore, requires ongoing assessment and further definition, such as greater insight into the persistence of *Salmonella* infection in wild birds and the routes by which they are exposed. The implementation of good farm biosecurity will help reduce this risk by minimizing opportunities for contact between wild birds and livestock such as poultry.

## Data availability statement

The whole-genome sequences were deposited in the National Center for Biotechnology Information (NCBI) National Library of Medicine under BioProject accession number PRJNA933150.

## Ethics statement

The animal study was reviewed and approved by Ethical Committee of the Animal Health Research Division at the Bangladesh Livestock Research Institute, Dhaka, Bangladesh (ARAC: 10/1/2018:01).

## Author contributions

MAS, RB, RC, and JFL: conceptualization. TC, RB, MSSarker, MSH, MSSagor, MAM, ASMAU, and MRK: methodology and investigation. MAS and RC: supervision. TC, MSSarker, RC, JFL, and MAS: data curation and formal analysis. MAS and RC: resources. RC, MAS, and TC: writing-original draft preparation. MAS, RB, RC, JFL, TC, MSSarker, MSH, MAM, ASMAU, and MRK: writing–review and editing. All authors contributed to the article and approved the submitted version.

## Funding

The study was conducted with the financial assistance of the Ministry of Fisheries and Livestock, Bangladesh and US CDC (Grant Number: 5 NU2GGH002077). RC and TC were supported by the UK FAO Reference Centre for Antimicrobial Resistance (which receives funding from the Department for Environment, Food & Rural Affairs and UK aid funding from the Department of Health and Social Care’s Fleming Fund).

## Conflict of interest

The authors declare that the research was conducted in the absence of any commercial or financial relationships that could be construed as a potential conflict of interest.

## Publisher’s note

All claims expressed in this article are solely those of the authors and do not necessarily represent those of their affiliated organizations, or those of the publisher, the editors and the reviewers. Any product that may be evaluated in this article, or claim that may be made by its manufacturer, is not guaranteed or endorsed by the publisher.

## Footnotes

^1^ http://www.ramsar.org/about-the-ramsar-convention

^2^ https://rsis.ramsar.org/ris/1031

^3^ https://www.graphpad.com/quickcalcs/confInterval1/

^4^ https://github.com/tseemann/nullarbor

^5^ https://github.com/tseemann/abricate
